# Reprocessable carbon fiber composites *via* disulfide-exchange epoxy vitrimers: experimental and molecular simulation insights

**DOI:** 10.1039/d6ra00851h

**Published:** 2026-03-31

**Authors:** Harsh Sharma, Nehal Kaushik, Songchang Liu, Rajkamal Anand, Nanda Gopal Sahoo, Gun Jin Yun, Sravendra Rana

**Affiliations:** a UPES, School of Engineering, Energy Acres Bidholi Dehradun Uttarakhand 248007 India gunjin.yun@snu.ac.kr; b TIET-VT, Centre of Excellence in Emerging Materials (CEEMS), Thapar Institute of Engineering and Technology Patiala Punjab 147004 India; c Department of Aerospace Engineering, Seoul National University Seoul 08846 South Korea srana@ddn.upes.ac.in; d Institute of Advanced Aerospace Technology, Seoul National University Gwanak-gu Gwanak-ro 1 Seoul 08826 Republic of Korea; e Prof. Rajendra Singh Nanoscience and Nanotechnology Centre, Department of Chemistry, Kumaun University D.S.B. Campus Nainital 263001 Uttarakhand India

## Abstract

The growing demand for carbon fiber-reinforced polymer (CFRP) composites in high-performance sectors such as wind energy and automotive underscores the urgent need for recyclable alternatives to traditional thermoset systems. Although disulfide-based epoxy vitrimers have shown promise for reprocessability, a clear correlation between dynamic bond exchange kinetics, network structure, and composite-level mechanical performance remains insufficiently understood. In this study, we present reprocessable and recyclable carbon fiber composites enabled by epoxy vitrimers dynamically crosslinked with 2,2′-dithiodibenzoic acid (DTBA). Among the tested formulations, the vitrimer with 2 wt% DTBA (EPD-2) exhibited optimal performance, combining high thermal stability (*T*_d5%_ = 396 °C), accelerated stress relaxation, and high self-healing efficiency (91%), indicating a balanced crosslink density and network mobility. Using vacuum-assisted resin infusion molding (VARIM), this EPD-2 matrix was integrated into carbon fiber composites (EPD-2-CF), which demonstrated high tensile strength (290 MPa), thermoformability, and shape recovery. The composites could be chemically degraded under controlled solvent-assisted conditions, enabling recovery of intact carbon fibers while preserving their structural integrity and enabling closed-loop fiber reclamation. Molecular dynamics (MD) simulations provided molecular-level validation of the composite mechanical response, with the simulated Young's modulus (2.74 GPa) in closely matching experimental results (2.69 GPa). Temperature-dependent creep simulations qualitatively reproduced experimental trends, revealing increased strain and delayed recovery from 130 °C to 170 °C due to activation of disulfide exchange mechanisms. This study establishes a vitrimer composite platform that correlates dynamic network design with composite viscoelastic behavior, advancing the development of recyclable high-performance CFRPS.

## Abstract 2

Carbon fiber-reinforced vitrimer composites containing dynamic bonds exhibit excellent self-healing and recyclability behavior. MD simulations were employed to gain atomic-level insights into elastic and viscoelastic behavior of vitrimer matrix..

## Graphical abstract

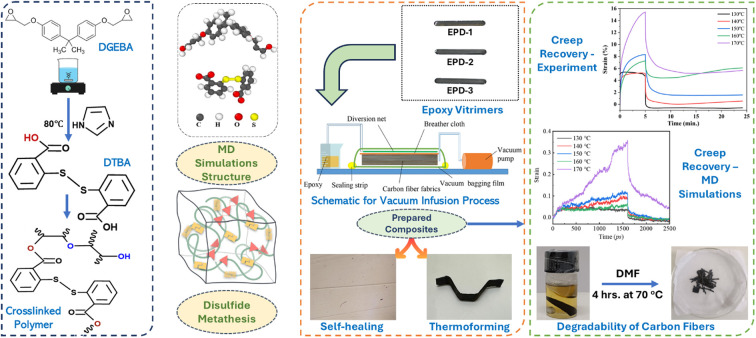

## Introduction

The deployment of lightweight carbon fiber-reinforced polymer (CFRP) composites in wind energy and automotive applications is projected to rise steadily, with an estimated compound annual growth rate of approximately 6%.^1^ This anticipated growth is attributed to their outstanding chemical and thermal resistance, as well as excellent mechanical properties. The growing demand for high-power wind turbines necessitates high-strength, large-scale, and lightweight blades. Upon reaching the end of their 20–25 year service life,^2^ wind turbine blades will generate a substantial amount of CFRP waste, projected to reach nearly 38 000 tons annually by 2030. Currently, only 5% of CFRP is recycled into lower-value secondary products.^3^ This scenario highlights the pressing need for effective strategies to manage CFRP waste and address the associated environmental challenges.^4,5^ Moreover, the crosslinked structure of thermoset CFRP poses significant challenges for recycling high-value carbon fibers. Traditional mechanical reprocessing methods are ineffective in recovering high-value carbon fibers^6^ and often generate noise, dust, and environmental pollution during the pulverization of CFRP into small particles. These particles are subsequently utilized as low-value fillers, fuel, or disposed of in landfills.^7^ On the other hand, chemical recycling enables the recovery of carbon fibers and precursors or monomers by breaking down the crosslinked thermoset structure.^8,9^ However, this process generally requires substantial quantities of organic solvents^10^ and may produce toxic by-products.^11,12^ Consequently, neither of the conventional recycling approaches can efficiently recover high-value carbon fibers. This limitation has prompted increasing interest in the development of reprocessable and degradable thermosets,^13–15^ which have the potential to effectively address the recycling challenges associated with conventional thermoset CFRPs.

As a solution to this, polymeric materials utilizing dynamic exchange bonds, known as vitrimers, have attention attracted significant in recent years. Vitrimers have progressed significantly since the pioneering work of Leibler *et al.* in 2011, which introduced ester exchange dynamic covalent bonding into polymer networks.^16–19^ These materials possess the characteristics of traditional thermosetting resins below their topological rearrangement temperature but can to alter their crosslinked structure when exposed to external stimuli such as heat, light, or chemical reagents, exhibiting thermoplastic-like behavior while retaining a permanent crosslinked network.^20–23^ Consequently, vitrimers combine the structural integrity of thermosets with the reprocessability of thermoplastics. Making them attractive candidates for sustainable polymer systems. Subsequent research has expanded vitrimer chemistry to various dynamic covalent bond exchange mechanisms including ester,^24^ disulfide,^25^ urea,^26^ and imine bonds.^27^ Among these dynamic chemistries, disulfide bond exchange mechanism has received considerable attention due to its relatively low activation energy, catalyst-free excahnfe reactions, and compatability with epoxy networks.^28,29^ The incorporation of disulfide bonds into epoxy matrices typically involves curing agents such as 2-aminophenyl disulfide (2-AFD) and 4-aminophenyl disulfide (4-AFD), where the amine functionality forms a densely crosslinked network with epoxy resins while the disulfide linkage provides dynamic reconfigurability to the polymer network.^30–32^ This dynamic behavior facilitates stress relaxation, self-healing, and recyclability in vitrimer systems. For example, Luzuriaga and coworkers^33^ synthesized an epoxy-vitrimer using 4-AFD as a curing agent; and the resulting composites exhibited an impressive flexural strength of 557 MPa and an interlaminar shear strength of 29 MPa while demonstrating good self-healing, recyclability, and malleability. Similarly, Si *et al.*^34^ prepared recyclable fiber-reinforced polymers (FRPs) containing aromatic disulfide bonds using a dual disulfide vitrimeric network. The developed vitrimer system could degrade in dilute dithiothreitol solution due to exchange reactions with external thiols, enabling efficient recovery and reuse of carbon fibers. Furthermore, Aranberri and co-workers,^35^ developed a dynamic epoxy resin suitable for pultrusion processes, enabling the fabrication of recyclable and reshapable CFRPs profiles. These composites could be mechanically recycled and reprocessed to produce second-generation composites reinforced with short carbon fibers while maintaining adequate mechanical performance. Recent studies have further demonstrated that integrating vitrimer chemistry into fiber-reinforced composites can significantly enhance their recyclability and multifunctionality. For instance, vitrimer-based epoxy matrices have been successfully used to fabricate reprocessable CFRP laminates capable of undergoing thermoforming and repair through dynamic bond exchange reactions.^27,36,37^ Similarly, recent investigations have reported vitrimer composites with improved stress relaxation and reshaping ability, allowing damaged composite structures to be repaired and reconfigured under thermal activation without compromising their mechanical integrity.^38–40^ Moreover, recent developments in recyclable vitrimer composites have highlighted the potential of dynamic covalent networks to enable closed-loop recycling of carbon fiber composites through selective chemical degradation of the matrix.^41,42^

Apart from the amine curing agents discussed above, a carboxylic acid-based curing agent, 2,2′-dithiodibenzoic acid (DTBA), has recently attracted significant attention due to its superior chemical and thermal stability compared to amine-based hardeners. This enhanced stability is attributed to the formation of strong ester linkages between the epoxy resin and DTBA, which are generally more stable than the amide linkages formed in amine-cured epoxy systems.^43^ Using this approach, Dutta and Karak^29^ synthesized an epoxy vitrimer exhibiting notable mechanical properties, including a tensile strength of 7.05 MPa, an elongation break of 148.80%, along with a healing efficiency of 71.48%. In addition, the prepared coatings demonstrated UV shielding capability, solvent resistance, and anti-oxidant properties while maintaining reprocessability and chemical degradability. Similarly, Huang and coworkers,^44^ developed a novel reprocessable thermosetting adhesive (RTA) based on DTBA, which exhibited excellent adhesive strength, self-healing capability, and recyclability. The dynamic hydroxyl ester and disulfide linkages facilitated healing with an efficiency of 91.8% at 180 °C, while the adhesive demonstrated a lap shear strength of 18.18 MPa on stainless steel surfaces. Extending this concept to sustainable materials, Mauro *et al.*^45^ reported a dual dynamic vitrimer network by curing epoxidized linseed oil with DTBA. The resulting vitrimer retained its mechanical properties even after multiple crushing and remolding cycles and could be rapidly dissolved in NaOH solution at 80 °C, confirming the enhanced reprocessing and degradation capability imparted by disulfide-containing dynamic networks. Recent investigations have also explored DTBA-based vitrimer matrices for recyclable polymer systems and composite structures, demonstrating improved thermal stability, dynamic exchange behavior, and recyclability of the resulting materials.^46,47^ Despite these advances in vitrimer chemistry, the application of DTBA-based vitrimer matrices in fiber-reinforced composite systems remains relatively underexplored, particularly in terms of correlating dynamic bond exchange behavior with composite-level mechanical performance and recyclability. Incorporating such vitrimers into FRPs has the potential to substantially enhance self-healing efficiency, recyclability, and thermoformability while maintain the structural integrity required for high-performance composite applications.

Therefore, in the present work, carbon fiber-reinforced vitrimer composites containing dynamic disulfide bonds were synthesized using DTBA as the curing agent *via* vacuum-assisted resin infusion molding (VARIM) process. The prepared vitrimer networks exhibit excellent self-healing efficiency, creep recovery, and recycling behavior. Thermal and dynamic properties of epoxy crosslinked networks were evaluated to determine their suitability for CFRPs fabrication. The mechanical performance, creep recovery behavior, and thermoformability of the resulting composites were systematically investigate. Additionally, the degradation mechanism of CFRP composites was studied to analyze the recovery of carbon fibers. To complement the experimental findings, molecular dynamics (MD) simulations were employed to provide atomic-level insights into the elastic and viscoelastic behavior of the vitrimer matrix, and the simulated mechanical properties were validated through a close agreement with experimental observations.

## Experimental section

### Materials

Diglycidyl ether of bisphenol-A (DGEBA) resin (340.41 g mol^−1^), 2,2′-dithiodibenzoic acid (DTBA) hardener (306.36 g mol^−1^), imidazole (IM) initiator (68.08 g mol^−1^), and dimethylformamide (DMF) were obtained from Sigma-Aldrich. A unidirectional CF cloth (3 K, 200 g m^−2^) was obtained from Composite Tomorrow, India. All products were commercially available and used without requiring further purification.

### Preparation of EPD epoxy vitrimers

**Scheme 1:**
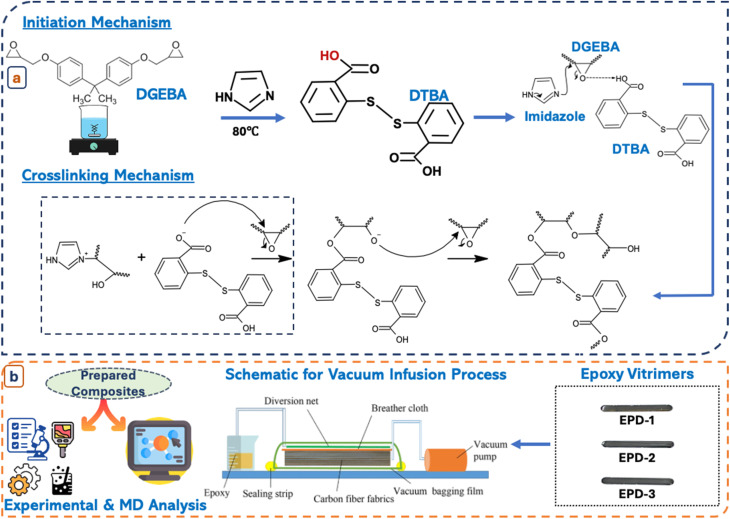
Schematic representation of (a) preparation of epoxy vitrimers; (b) preparation of vitrimer composites.

The epoxy monomer was heated to 80 °C, followed by the addition of 1 wt% of the initiator, imidazole. Subsequently, the required amounts of the hardener, 2,2-dithibenzoic acid were incorporated into the solution and stirred at the same temperature for 10 minutes. The DTBA and imidazole concentration represents the weight percentage of curing agent relative to the epoxy resin (as detailed in Table 1 and illustrated in Scheme 1(a)). The mixture then underwent a curing reaction, which initiates with a nucleophilic attack on the highly strained epoxide ring by imidazole, leading to the ring opening. The intermediate then interacts with an acidic –COOH group present in the system, abstracting a proton and generating a carboxylate ion. This stabilization allows for further reactivity with another epoxide moiety. The carboxylate anion undergoes a nucleophilic reaction with another epoxide group, leading to the formation of ester linkages. This reaction repeats, resulting in an extensive crosslinked polymer network where multiple epoxide groups are converted into ester crosslinks.^48^ The prepared mixture was poured into molds and cured sequentially at 80 °C for 2 hours, 120 °C for 2 hours, and 150 °C for 5 hours. DTBA was selected as the curing agent due to its ability to introduce dynamic disulfide bonds into the epoxy network while simultaneously forming thermally stable ester linkages with epoxy groups. Different concentrations of DTBA (1–3 wt%) were investigated to optimize the dynamic exchange behavior and mechanical performance of the vitrimer network. Among the investigated formulations, the EPD-2 composition demonstrated the most balanced combination of thermal stability, creep relaxation behavior, and self-healing efficiency and was therefore selected for composite fabrication.

**Table 1:** Different proportions of epoxy, initiator and hardener to prepare epoxy thermosets

S. no.	Sample ID	DGEBA (g)	IM (wt%)	DTBA (wt%)
1	EPD-1	2	1	1
2	EPD-2	2	1	2
3	EPD-3	2	1	3

It is worth noting that conventional epoxy resins, typically derived from bisphenol A, possess a rigid and well-defined aromatic backbone that results in a dense and uniform molecular architecture with a high crosslinking density. This intrinsic rigidity, coupled with the well-controlled reactivity of epoxy groups, enables dithiodibenzoic acid (DTBA) to effectively facilitate curing even at relatively low concentrations, as the uniformly distributed reactive sites promote efficient network formation without the need for excess DTBA. In contrast, bio-based epoxy systems generally feature aliphatic backbones with a non-uniform distribution of functional groups, leading to reduced inherent rigidity. Consequently, higher DTBA concentrations are required to achieve comparable crosslinking density and network stability. However, excessive DTBA loading can introduce additional complexities, as unreacted carboxylic acid groups may participate in hydrogen bonding either among themselves or with hydroxyl groups generated during partial curing, thereby diminishing the efficiency of network formation. Therefore, careful optimization of DTBA concentration is essential and must be tailored to the specific epoxy matrix employed. For comparison, epoxy thermosets containing higher DTBA loadings (5, 10, and 15 wt%) were also prepared following the same synthesis protocol, as illustrated in Fig. S1, and their solubility behavior was subsequently evaluated, as shown in Fig. S2.

### Preparation of EPD-CF composite

Carbon fiber vitrimer composites were fabricated using a vacuum-assisted resin infusion molding system. Four layers of unidirectional carbon fibers, each cut to dimensions of 11 cm × 11 cm × 0.1 mm, were sequentially arranged on a steel plate and covered with peel ply, infusion mesh, and vacuum bagging film. Simultaneously, epoxy resin solutions with varying DTBA hardener concentrations (EPD-1, 2, 3) were prepared by mixing at 80 °C. After thorough mixing, the solution was degassed and infused into the vacuum bag through a PVC hose, ensuring complete saturation of the carbon fibers. The infused composite plate was then pre-cured in a vacuum oven at 120 °C for 2 hours, followed by post-curing at 150 °C for 5 hours, as illustrated in Scheme 1(b).

### Characterization

To comprehensively evaluate the vitrimer system, a combination of thermal analysis (TGA, DSC), mechanical characterization, creep recovery studies, recyclability assessment, and molecular dynamics simulations were employed. All experiments were conducted under controlled conditions assuming uniform sample composition and consistent experimental parameters to ensure reliable comparison of thermal, mechanical, and self-healing performance. FTIR spectra were collected on a PerkinElmer spectrophotometer using attenuated total reflection (ATR) mode. Data acquisition involved 16 scans from 4000 to 400 cm^−1^ at a resolution of 4.0 cm^−1^. The glass transition temperature (*T*_g_) of the cured samples was determined using differential scanning calorimetry (DSC) on a Hitachi High-Tech Science DSC7020 instrument. Approximately 8–10 mg of each sample was placed in a pierced aluminium pan and analyzed under a N_2_ flow of 50 mL min^−1^. The thermal stability of the cured samples was investigated using thermogravimetric analysis (TGA) on a Hitachi High-Tech Science STA200 instrument. Approximately 10 mg of each sample was subjected to thermal degradation from 30 °C to 800 °C at a heating rate of 10 °C min^−1^ under a continuous flow of N_2_ at 100 mL min^−1^. To assess the material properties, gel content and swelling ratio were determined. Dry samples (approximately 30 mg, initial weight *W*_0_) were extracted in 5 mL of acetone for 24 hours. Following extraction, the samples were weighed (*W*_1_), then dried in an oven at 40 °C for 5 hours and reweighed (*W*_2_). The gel content and swelling ratio were calculated using the following eqn (1) and (2):1
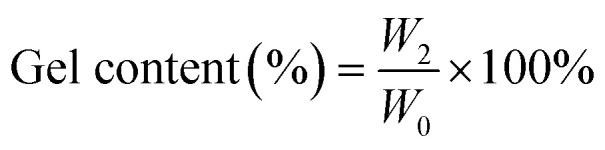
2
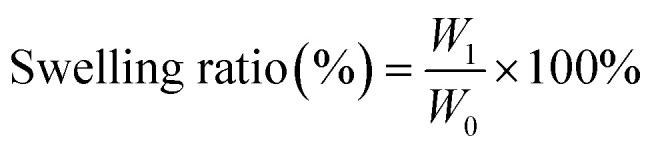


Thermomechanical properties were measured using a TA instruments TMA-Q400 equipped with a three-point bending clamp. Rectangular samples of about 15 mm × 5 mm × 0.5 mm were analyzed at 0.50 Hz, 0.1% strain, and from 40 to 120 °C at 3 °C min^−1^. Stress–strain experiments were performed in strain ramp mode with a constant preload of 0.02 N to maintain contact between the probe and the sample during the measurement. Strain measurements were performed at a constant temperature of 40 °C. Stress relaxation tests were conducted by first equilibrating samples at temperatures around the *T*_g_. A constant strain of 1% (within the linear viscoelastic region) was then applied to the sample, and the subsequent stress decay was monitored as a function of time. This procedure was repeated at 10 °C intervals up to a maximum temperature of 90 °C. Creep recovery properties were investigated by applying a stress of 0.3 MPa for 5 min and at 70 °C, followed by immediate stress release and a 20 min recovery period. This procedure was repeated at 10 °C intervals, culminating at a final temperature of 110 °C. Creep recovery was evaluated by measuring the strain recovery after removal of the applied stress at elevated temperature.

The self-healing capability of the cured sample was assessed by introducing a scratch using a razor blade. Subsequently, the samples were subjected to thermal treatment in an oven at 80 °C for durations of 20 and 40 min. The healing progress of the scratch was then observed using an Olympus BX51 optical microscope. The healing efficiency is considered to be the ratio of the healed scratch width to the original scratch width of the samples, which is calculated by the following eqn (3):3
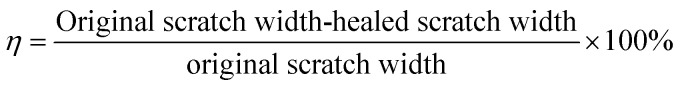
where *η* represents the healing efficiency.

Rectangular specimens of carbon fiber vitrimer composites, with dimensions of 100 mm × 10 mm × 2 mm, underwent tensile strength testing using a universal testing machine. The tests were performed at a constant temperature of 26 °C and a strain rate of 2 mm min^−1^. To ensure data reliability, each composition was tested three times. To assess the thermoforming ability of the vitrimer composite, the EPD-2-CF sample, along with the mould, was placed in a preheated oven at 120 °C for 10 min, together with the weights used for reshaping.

To investigate the degradation process of the EPD-2-GF composite and the recovery of carbon fibers, the composite samples were treated in dimethylformamide (DMF) at 70 °C for 4 hours. The degradation products were characterized through Fourier Transform Infrared (FTIR) spectroscopy. The structural analysis of both the original and recovered carbon fibers was performed using X-ray diffraction (XRD) with a D8 ADVANCE ECO Bruker system, operating at 40 kV, scanning over a 2θ range of 0.5° to 80°, and maintaining a scanning speed of 5° min^−1^. Additionally, the surface morphology of virgin and recycled carbon fibers was analyzed with a ZEISS scanning electron microscope (SEM) under an accelerating voltage of 20 kV.

## Simulation methodology

### Simulation details

In this study, all MD simulations were conducted using Large-scale Atomic/Molecular Massively Parallel Simulator (LAMMPS).^49^ The Polymer Consistent Force Field (PCFF) was employed to describe the interactions within the system, as it is widely used for investigating the properties of polymer nanocomposites.^50,51^ Non-bonded interactions were calculated using a cutoff radius of 9.5 Å, and the Ewald summation method with an accuracy of 0.0001 kcal mol^−1^ was applied. Throughout the simulations, the equations of motion were integrated using the Verlet algorithm^52^ with a timestep of 1 fs.

### Modelling

**Fig. 1:**
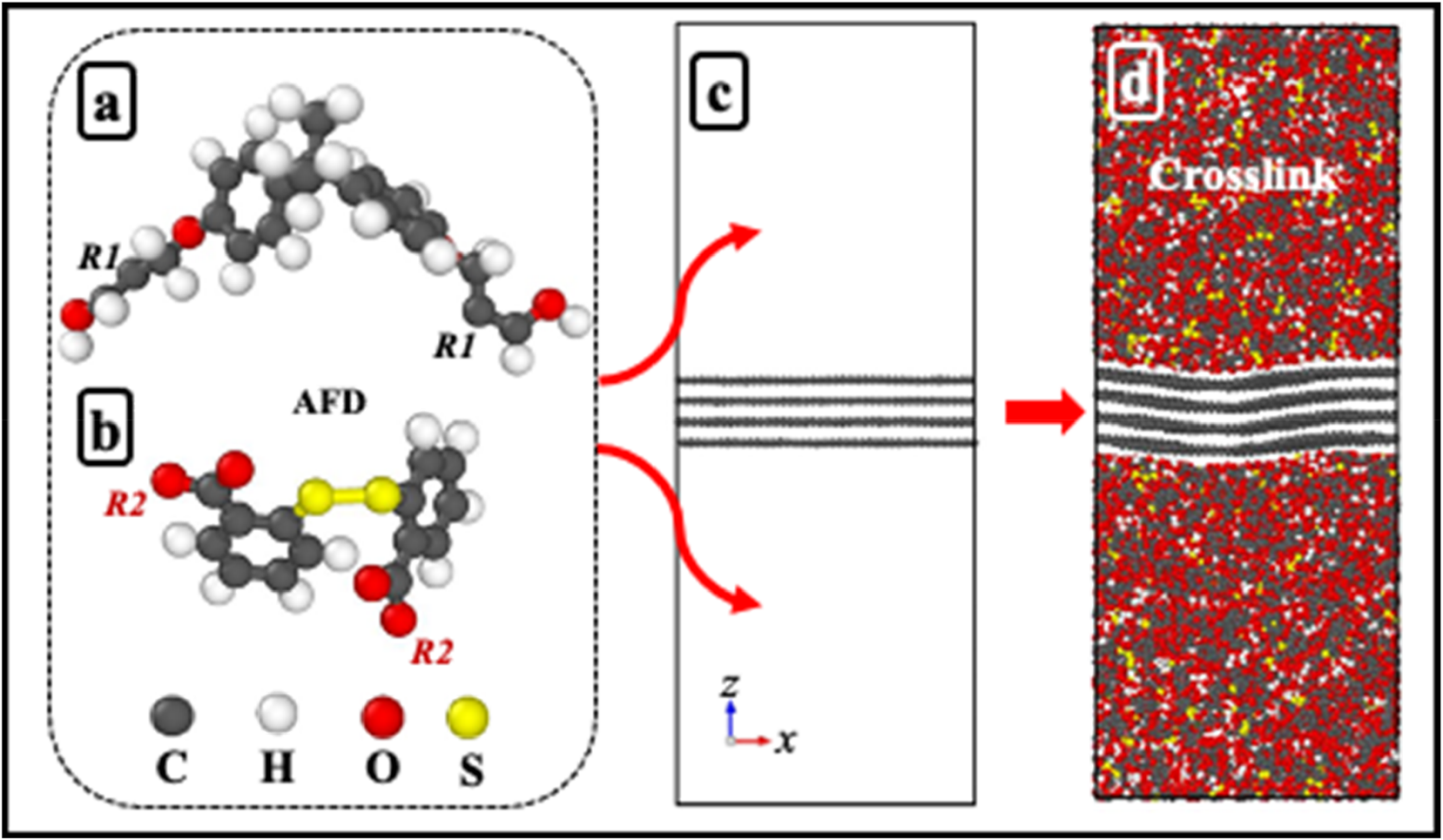
Optimized structures of (a) DGEBA and (b) AFD. R1 and R2 are the reactive C atoms in DGEBA and the reactive O atoms in AFD. (c) Carbon layers and (d) crosslink inserted carbon layers of simulations box.

Due to the limitations of MD simulations in terms of scale, constructing a complete carbon fiber model is challenging. A common alternative is to represent the outer layers of carbon fiber using parallel carbon sheets, which can effectively capture the enhancement effects of carbon fiber on various material properties.^53,54^ As shown in Fig. 1, 4 layers of carbon were placed within a simulation box measuring 47 × 49 × 129 Å, with periodic boundary conditions applied in all three directions. Subsequently, the epoxy matrix was introduced into the vacuum regions. Two common methods for constructing crosslinked epoxy networks are the script crosslinked method^55^ and the representative molecule method.^56^ Although the latter simplifies the process by directly constructing crosslinked representative units, it cannot simulate the dynamic crosslinking process. To enhance the reliability of the simulation results, the script crosslinked method was chosen in this work. Specifically, 126 DGEBA and AFD molecules were placed in the top and bottom voids. New bonds were formed between atomic pairs within the reaction cutoff distance of 8 Å, resulting in a crosslinking density of 90% in the final structure. Finally, Bond Exchange Reaction (BER) equilibration was performed on the target structure by iteratively breaking and reforming disulfide bonds using a cyclic script during the dynamic simulation. Detailed procedures can be found in our previous work.^50^ To minimize residual stress within the final simulation box, additional annealing simulations were conducted. The system was first relaxed at 300 K for 50 ps under the NPT ensemble, then heated to 600 K and equilibrated for 50 ps, followed by cooling back to 300 K with a final relaxation for 50 ps. The resulting system density was calculated to be 1.27 g cm^−3^, which is in good agreement with the experimental value of 1.25 g cm^−3^, confirming the validity of our model.

### Uniaxial tensile simulation

In the tensile simulation, a load was applied in the *z*-direction, which is perpendicular to the carbon sheets. The structure was stretched at a constant strain rate of 5 × 10^8^ s^−1^, while the *x* and *y* directions were allowed to contract freely to maintain a pressure of 0 bar. The stresses of the system were calculated by Virial's theorem^57^ with the following expression:
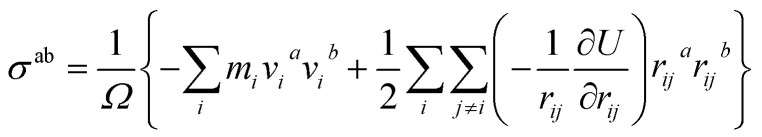
where *σ*^ab^ is the global stress tensor, *Ω* represents the volume of the structure, *r*_*ij*_^*a*^ is the *a*th component of the relative position vector between atom *i* and *j*. *m*_*i*_ and *v*_*i*_ stand for the atomic mass and velocity of atom *i*. The strain equation is (*l* − *l*_0_)/*l*_0_, where *l*_0_ and *l* denote the initial and instantaneous values of the simulated box length, respectively.

## Results and discussions

### Fundamental characterization of the EPD vitrimers

**Fig. 2:**
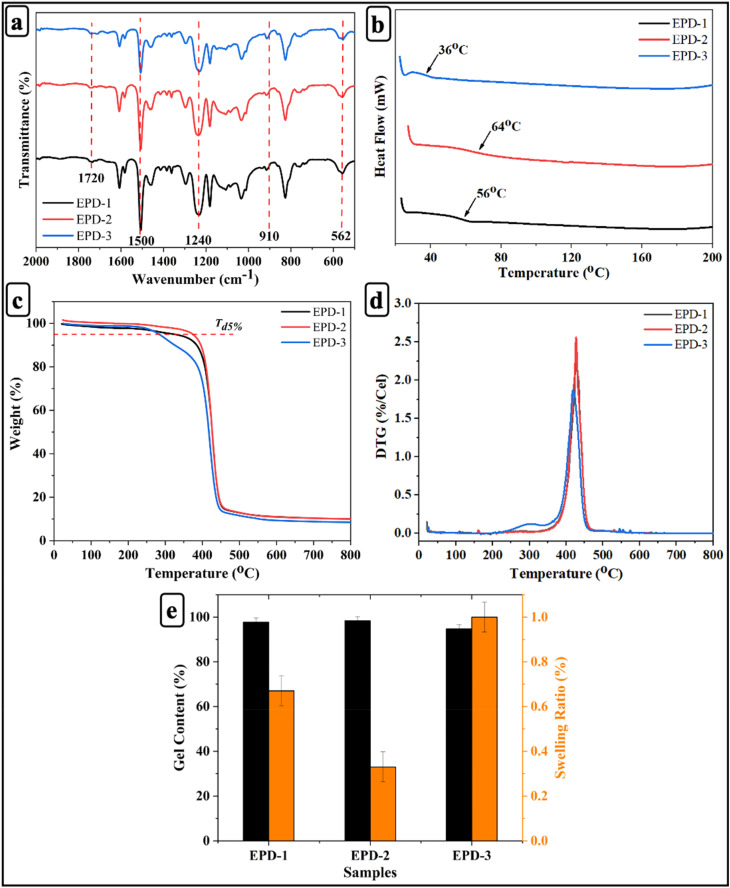
(a) FT-IR spectra; (b) DSC thermograms; (c) TGA curves; (d) DTG curves; (e) gel content and swelling ratio of EPD-1, EPD-2, and EPD-3 epoxy vitrimers.

The fabrication of crosslinked networks with dynamic properties was carried out following the methods outlined in previous studies.^4^ The FTIR spectra of EPD-1, EPD-2, and EPD-3 exhibit characteristic absorption bands corresponding to the formation of the vitrimer network Fig. 2(a). The band observed around ∼1720 cm^−1^ corresponds to the C=O stretching vibration of ester groups, confirming the reaction between epoxy groups and DTBA. Peaks near ∼1500 cm^−1^ are attributed to aromatic C=C vibrations originating from the epoxy backbone. The absorption band in the ∼1240 cm^−1^ region corresponds to C–O–C stretching vibrations, indicating the formation of ester linkages in the crosslinked network. The peak at ∼910 cm^−1^ corresponds to the characteristic epoxide ring vibration, while the band at ∼562 cm^−1^ is assigned to S–S stretching, confirming the incorporation of disulfide bonds into the vitrimer structure.^58^ Disulfide stretching vibrations are generally reported in the 450–550 cm^−1^ region, although slight shifts may occur depending on the molecular environment and structural interactions within the polymer network.^59^

The curing behavior of EPD mixtures was investigated using DSC. EPD-1, EPD-2, and EPD-3 samples displayed exothermic peaks at 56 °C, 64 °C, and 36 °C, respectively (Fig. 2(b)), confirming the occurrence of curing reactions between epoxy and carboxyl groups within the feedstock. A three-stage curing protocol was employed: 80 °C for 2 hours, followed by 120 °C for 2 hours, and finally 150 °C for 5 hours. This multi-step process was designed to ensure complete curing while minimizing the risk of material degradation.

The thermal degradation behavior of the EPD mixtures was investigated (Fig. 2(c) and (d)). The TGA results revealed that all samples exhibited high thermal stability up to ∼300 °C, with negligible weight loss in this range. The degradation temperature corresponding to 5% weight loss (*T*_d5%_) was highest for EPD-2, highlighting the optimal crosslink density achieved with 2 wt% DTBA. Moreover, the DTG curves demonstrated a single major decomposition peak for all systems, centred around 400–450 °C, attributed to the breakdown of the crosslinked epoxy network. EPD-2 exhibited a slightly higher peak temperature (*T*_max_) compared to EPD-1 and EPD-3, confirming its enhanced thermal stability. The reduced stability of EPD-1 and EPD-3 can be attributed to either insufficient or excessive concentrations of the curing agent, resulting in either incomplete crosslinking or structural defects.^60^

The gel content and swelling ratio provide valuable insights into the extent of crosslinking within the network.^61^ As illustrated in Fig. 2(e), the EPD-2 epoxy vitrimer exhibits high gel content and a low swelling ratio, which corroborates the findings of FT-IR and DSC analysis regarding the curing behavior of the samples.

### Dynamic characterization of the EPD vitrimers

**Fig. 3:**
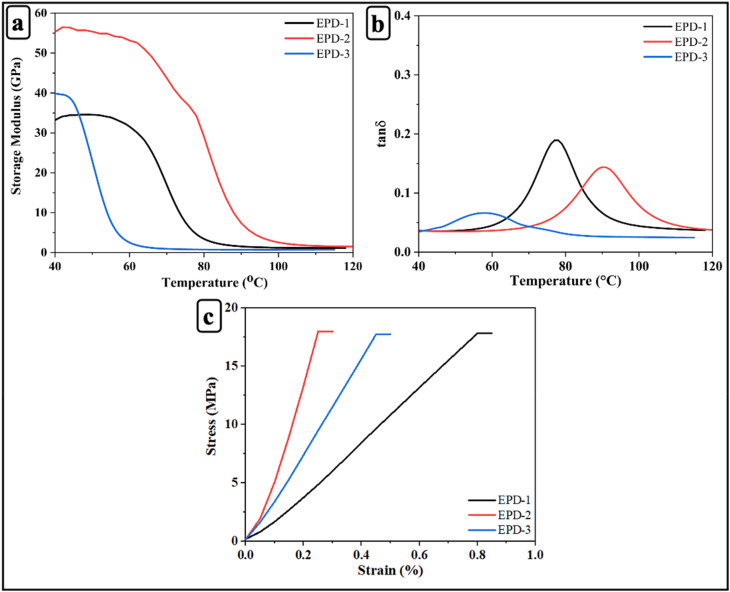
(a) Storage modulus; (b) tan *δ*; (c) stress–strain relationship of EPD-1, EPD-2, and EPD-3 epoxy vitrimers.

Fig. 3(a) illustrates the storage modulus *vs.* temperature curves for the epoxy vitrimers. The storage modulus values were obtained from TMA performed in three-point bending mode, where the modulus was calculated from the load-deflection response under controlled heating conditions. At 40 °C, EPD-2 exhibited the highest initial storage modulus (55 GPa), indicative of superior stiffness and an optimally crosslinked network compared to EPD-1 (40 GPa) and EPD-3 (33 GPa). A sharp decline in storage modulus with increasing temperature signifies the *T*_g_, which was highest for EPD-2, suggesting optimal thermal-mechanical stability attributed to balanced crosslinking. Conversely, lower *T*_g_ values were observed for EPD-1 and EPD-3, likely due to lower crosslink density and a less robust network structure, respectively. Above 100 °C, all systems transitioned to a rubbery state, as evidenced by a substantial reduction in storage modulus values. These results collectively demonstrate that EPD-2 exhibits the most efficient crosslinking, leading to enhanced mechanical stiffness and improved thermal resistance.

The *T*_g_ was determined by identifying the peak temperature of the tan *δ* curve.^62,63^Fig. 3(b) and Table 2 summarize the results. Tan *δ* analysis revealed that EPD-2 exhibited the highest *T*_g_ (92 °C), followed by EPD-1 (78 °C) and EPD-3 (57 °C), suggesting that the 2 wt% DTBA curing concentration optimizes crosslink density and enhances thermal-mechanical stability. The peak height of tan *δ* was highest for EPD-1, indicative of greater polymer chain mobility due to insufficient crosslinking, while EPD-2 demonstrated moderate damping, characteristic of a well-structured network. EPD-3 exhibited the lowest peak height, attributed to restricted chain mobility resulting from potential over-curing or excessive crosslinking. Furthermore, EPD-2 displayed the narrowest tan *δ* peak, suggesting a more homogeneous network structure, whereas broader peaks were observed for EPD-1 and EPD-3 indicating network heterogeneity.

**Table 2:** The detailed results of thermal, dynamic and self-healing properties of samples

Sample name	*T*_g_ (°C)		Flexural strength (MPa)	Storage modulus (GPa)	*T*_d5%_ (°C)	Gel content (%)	Swelling ratio (%)	Self-healing efficiency (%)
	TMA	DSC						
EPD-1	77.5	56	17.8	33	384	97.7	0.67	84
EPD-2	90.3	64	18.5	55	396	98.3	0.33	91
EPD-3	57.8	36	17.7	40	325	94.7	1	86

Three-point bending tests conducted at 40 °C in strain ramp mode were used to assess the stress–strain behavior of the epoxy vitrimers. Fig. 3(c) illustrates the flexural stress–strain curves for EPD-1, EPD-2, and EPD-3. EPD-2 exhibited the highest elastic modulus and stiffness, as evidenced by the steepest slope in the curve, achieving a maximum stress of 17 MPa at a strain of around 0.2%. This superior performance is attributed to optimal crosslinking at 2 wt% DTBA. In contrast, EPD-1, with a lower DTBA concentration, displayed reduced strength due to insufficient crosslinking. EPD-3 exhibited the lowest mechanical strength (10 MPa), likely due to irregularities in the network. EPD-1 and EPD-3 exhibited higher failure strains (0.8% and 0.4%, respectively), suggesting greater ductility but reduced rigidity.

### Characterization of the EPD vitrimers network rearrangement

**Fig. 4:**
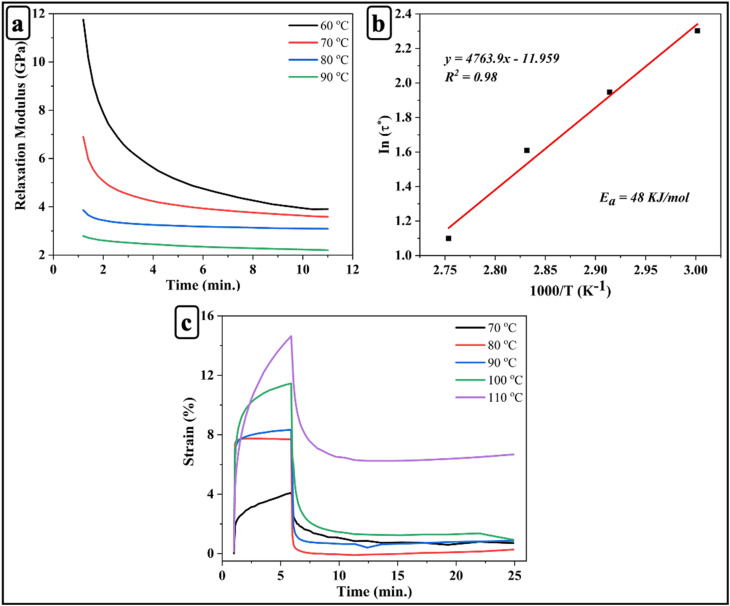
(a) Stress-relaxation of EPD-2 at different temperatures; (b) In(*τ*) *versus vs.* 1000/*T* plot for EPD-2 epoxy vitrimer; (c) creep curves of EPD-2 at different temperatures.

Stress relaxation tests were employed to investigate the topological rearrangement of epoxy vitrimers incorporating dynamic disulfide bonds. The relaxation time (*τ*), defined as the time for stress or modulus to decay to 1/*e* (36.7%) of its initial value.^28,60^ The temperature dependence of *τ* for EPD-2 is illustrated in Fig. 4(a). Increasing the temperature from 60 °C to 90 °C resulted in a decrease in *τ* from 480 s to 120 s. This observed reduction is attributed to the increased exchange rate of the dynamic disulfide bonds at elevated temperatures, which, in conjunction with enhanced chain segment mobility, facilitates the rearrangement process.^64^ The relaxation time of EPD-2 exhibits Arrhenius-type behavior:^65^4
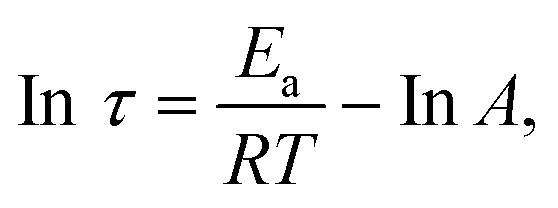
where *τ* represents the relaxation time, *T* is the absolute temperature, *E*_a_ is the activation energy for the dynamic bond exchange reaction, *R* is the universal gas constant, and *A* is the pre-exponential factor. As shown in Fig. 4(b), the linear fit to the data (*y* = 4763.9*x* − 11.95) yielded an activation energy (*E*_a_) of 48 kJ mol^−1^. The calculated activation energy falls within the range typically reported for disulfide exchange-based vitrimer networks,^66^ indicating efficient dynamic bond exchange that facilitates network rearrangement, stress relaxation, and recyclability at elevated temperatures.^25^

Moreover, the creep recovery behavior of EPD-2 was also evaluated. Fig. 4(c) illustrates the creep behavior, which is attributed to the temperature-accelerated dynamics of the disulfide bonds. Increasing the temperature from 70 °C to 110 °C resulted in a progressive increase in the slope of the creep deformation, indicating accelerated dynamic behavior.^67,68^ The observed decrease in the deformation recovery rate is likely due to slippage between polymer chains. These pronounced dynamic properties of the disulfide bonds at elevated temperatures suggest the potential for recyclability and reprocessability of the crosslinked epoxy vitrimer.

### Degradability and self-healing of the EPD-2 vitrimer

**Fig. 5:**
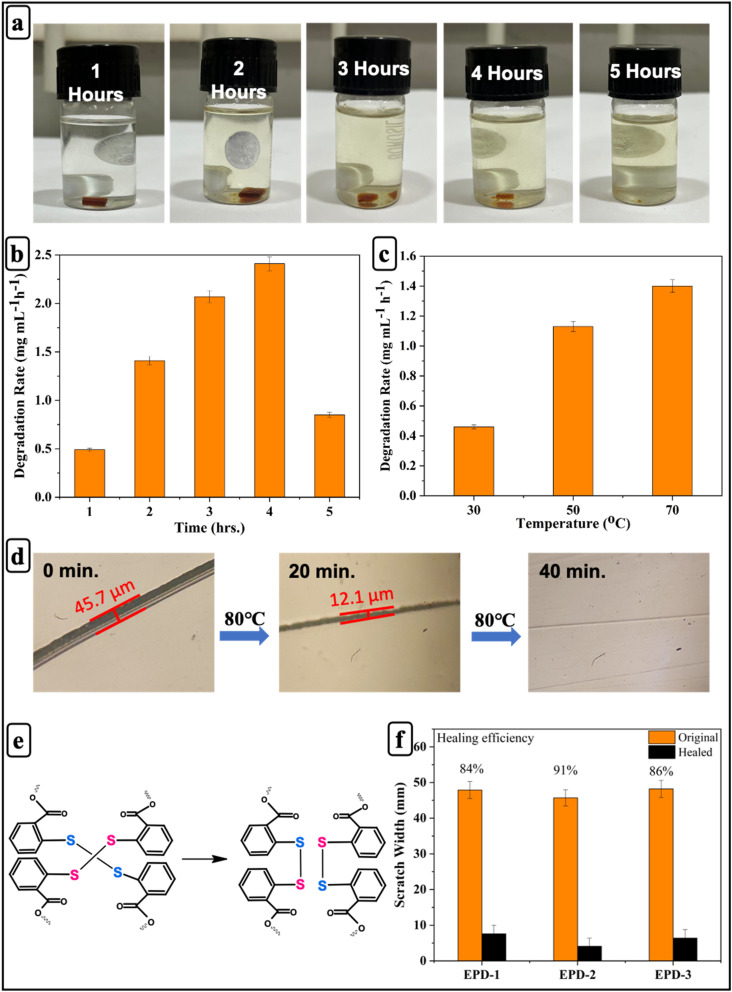
(a) Degradation process at different times in 10 mL DMF solution; (b) degradation rates at different times; (c) degradation rates at different temperatures; (d) self-healing test of EPD-2 epoxy vitrimer; (e) schematic of the dynamic disulfide exchange mechanism enabling vitrimer self-healing; (f) healing efficiency of epoxy vitrimers.

Covalently crosslinked networks containing disulfide bonds exhibit degradation behavior under mild acidic conditions,^69^ where disulfide bonds break down into hydroxyl and sulfhydryl groups,^70^ as illustrated in Fig. 5(a). Accordingly, the degradation performance of the EPD-2 epoxy vitrimer can be evaluated by varying time and temperature.

First of all, to assess the degradation behavior of the EPD-2 sample (∼30 mg), it was immersed in DMF solution for varying time. As illustrated in Fig. 5(b), the EPD-2 sample undergoes gradual degradation over time, causing the solution to darken progressively and leading to complete dissolution within 5 hours. Moreover, the degradation rate was evaluated based on the weight loss per unit time. An initial degradation rate of 0.49 mg mL^−1^ h^−1^ was observed at 1 hour, which increased to 1.41 mg mL^−1^ h^−1^ at 2 hours and further to 2.07 mg mL^−1^ h^−1^ at 3 hours, reaching a maximum of 2.41 mg mL^−1^ h^−1^ at 4 hours. Interestingly, at 5 hours, the degradation rate declined to 0.85 mg mL^−1^ h^−1^, likely due to the limited availability of fresh DMF to dissolve the degradation products, which may reduce solvent diffusion and slow the removal of degraded polymer fragments from the composite matrix.^71^

Furthermore, the degradation performance of EPD-2 vitrimer containing disulfide bonds has been assessed at different temperatures and depicted in Fig. 5(c). At 30 °C, the degradation rate is approximately 0.4 mg mL^−1^ h^−1^, indicating a relatively slow degradation process at lower temperatures. As the temperature increases to 50 °C, the degradation rate rises significantly to around 1.1 mg mL^−1^ h^−1^, suggesting that the dynamic covalent bond exchange is more active at moderate temperatures. At the highest tested temperature of 70 °C, the degradation rate reaches approximately 1.4 mg mL^−1^ h^−1^, showing a pronounced acceleration of the degradation process. This trend confirms that the vitrimer undergoes thermally activated bond rearrangements, where higher temperatures enhance the mobility and cleavage of dynamic disulfide linkages.

Due to the exchangeable bond reaction (Fig. 5(e)), epoxy vitrimers possess the ability to self-heal under external stimuli. An optical microscope was used to observe the healing process from a macroscopic perspective. The optical images of samples healed at 80 °C for 20 and 40 min are shown in Fig. 5(d). Initially, the crack is visible. After 20 minutes of healing, the crack becomes smaller, and its trace appears lighter. After 40 minutes, the crack traces are barely noticeable. To confirm the self-healing performance, the healing efficiency of different samples was quantified using the method described in the characterization section. As shown in Fig. 5(f), among all the samples, EPD-2 exhibits the highest healing efficiency (91%) compared to EPD-1 and EPD-3. These findings suggest that incorporating disulfide bonds into the polymer network enhances dynamic bond exchange reactions and molecular chain rearrangements, enabling the EPD-2 epoxy vitrimer to efficiently repair cracks.

### Dynamic, mechanical, and thermoforming behavior of EPD-2-CF composites

**Fig. 6:**
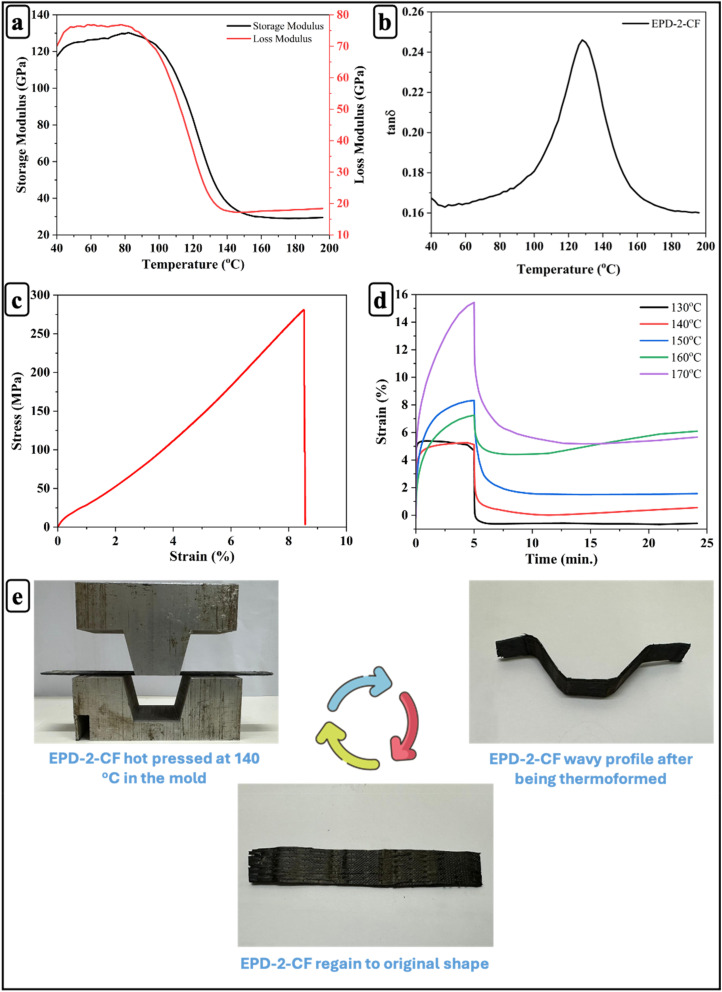
(a) Storage modulus; (b) tan *δ*; (c) Stress-strain relationship; (d) creep curves at different temperatures; (e) thermoforming test of EPD-2-CF vitrimer composite.

EPD-2 was chosen as the CFRP matrix due to its excellent mechanical properties and rapid stress relaxation, which enable the fabrication of CFRPs with both high dynamic and mechanical performance. Fig. 6(a)–(c) illustrate the dynamic properties of EPD-2-CF. The storage modulus (∼120 GPa), *T*_g_ (130 °C), and tensile strength (290 MPa) of EPD-2-CF were higher than those of EPD-2. This demonstrates that CF effectively enhances the dynamic properties of EPD-2 epoxy vitrimers.

The creep behavior of EPD-2-CF composites was examined at temperatures ranging from 130 °C to 170 °C (Fig. 6(d)). The results demonstrate a clear temperature dependence, with higher temperatures resulting in a significant increase in creep strain. This behavior is closely related to the thermal activation of the vitrimer network and the glass transition characteristics of the matrix. As the temperature approaches and exceeds the glass transition region, increased segmental mobility facilitates disulfide bond exchange and network rearrangement, leading to enhanced creep deformation. In the composite system, the presence of rigid carbon fibers restricts polymer chain mobility and limits matrix deformation due to their high stiffness and load-bearing capability.^72,73^ Consequently, higher temperatures are required to activate sufficient molecular mobility and dynamic bond exchange, which governs the temperature-dependent viscoelastic response of the EPD-2-CF composites. At 130 °C, the composite exhibited minimal creep, reaching a strain of approximately 0.5% after 5 min. As the temperature increased to 170 °C, the disulfide exchange reaction was fully activated, resulting in a strain of approximately 14%. Upon removal of the applied stress, the strain rapidly recovered and then stabilized, indicating that the disulfide exchange reaction governs the deformation behavior of EPD-2-CF.

Fig. 6(e) illustrates the thermoforming process of EPD-2-CF vitrimeric composites, which utilizes the reversible nature of disulfide bonds at temperatures above the material's *T*_g_. Initially, the EPD-2-CF composite is hot-pressed in a mold at 140 °C, a temperature exceeding its *T*_g_, enabling the dynamic exchange of disulfide bonds. This exchange allows the normally rigid material to become pliable and flow under applied pressure, conforming to the shape of the mold. Upon cooling below *T*_g_ while still in the mold, the dynamic bond exchange slows significantly, effectively “freezing” the newly formed shape. As a result, the EPD-2-CF composite assumes a permanently deformed, wavy profile, demonstrating successful thermoforming. When the thermoformed wavy sample is reheated to 100 °C, the mobility of the disulfide bonds is restored, allowing the material to relax and return to its original flat shape. This recovery highlights the reversible nature of disulfide bonds and underscores the potential of these materials for applications requiring reprogrammable shapes or repairability.

### MD simulations of uniaxial tensile and creep behavior

**Fig. 7:**
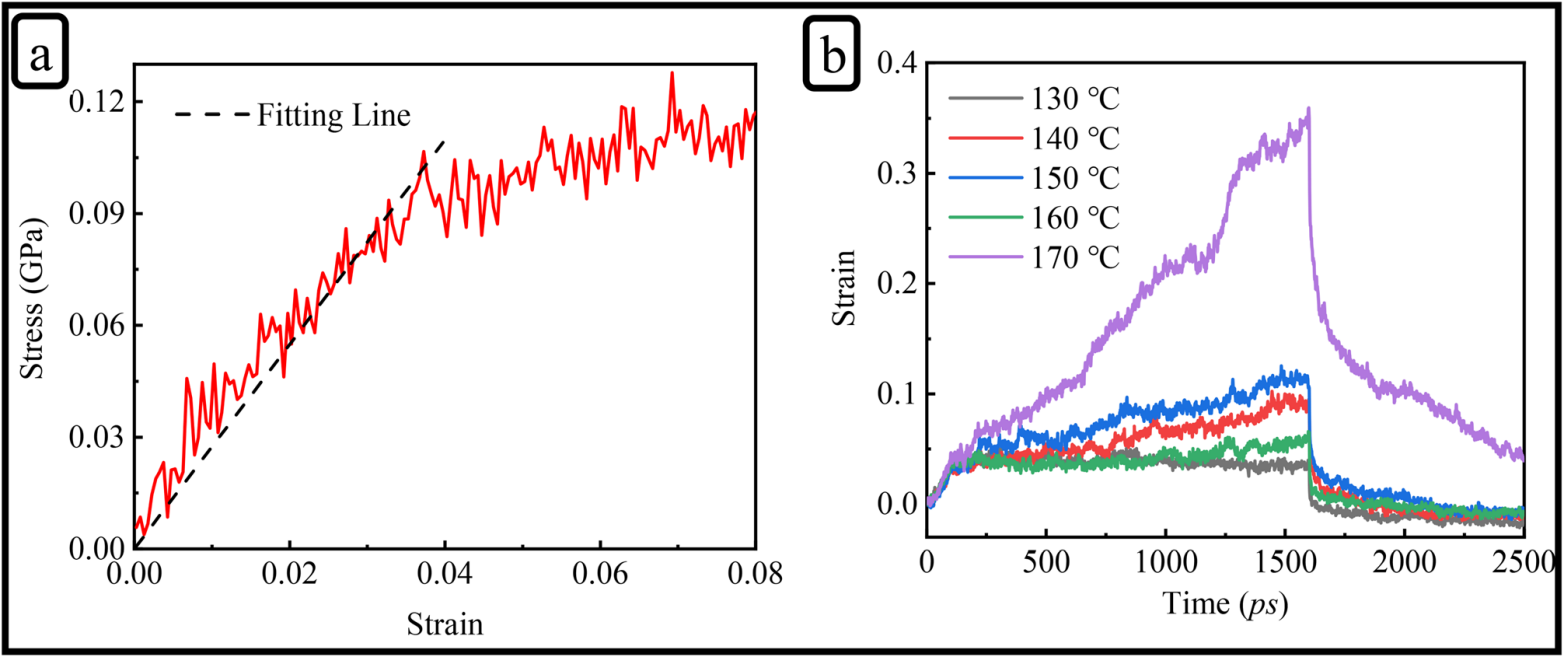
(a) Tensile stress–strain response of vitrimer-based CFRP, with the black dashed line indicating the fit used to determine Young's modulus. (b) Creep behavior at various temperatures showing strain evolution over time.

To support and rationalize the experimental observations, MD simulations were carried out to explore the underlying molecular mechanisms governing the mechanical response of the vitrimer-based CFRP system. Fig. 7(a) shows the stress–strain curves within the strain range of 0 to 0.08 at room temperature. By fitting the curves, the Young's modulus obtained from MD simulations is 2.74 GPa, which is very close to the experimental value of 2.69 GPa, further confirming the accuracy of the simulations. As like experimental analysis, we did not observed sudden drop in stress, even when the strain was further increased in simulation. This discrepancy is understandable, as the PCFF force field cannot simulate bond breaking during the simulation. Consequently, the system can only relax stress through molecular chain sliding or bending, making it impossible to simulate the initiation and rapid propagation of microcracks.^50,51^ In contrast, the sudden drop in stress observed in real materials is typically associated with the formation of cracks. Therefore, directly comparing the yield stress and strain between the simulation and experimental results is challenging. Moreover, differences in strain rates, fabrication defects present in experimental samples, and other factors may also contribute to the discrepancy between simulation and experimental data. Nonetheless, the reliability of model can still be validated by comparing the Young's modulus in the linear elastic region.

To simulate the creep behavior, we studied the strain-time curve across a temperature range of 130–170 °C and the corresponding strain–time curves are presented in Fig. 7(b). Each temperature, a constant pressure of 80 MPa was applied in the *z*-direction of structure (see Fig. 1(d)) under the NPT ensemble to stretch. The simulations revealed a clear T-dependent response. Up to 160 °C, the vitrimer network exhibited stable deformation and moderate strain recovery, suggesting this *T* as a potential threshold for optimal mechanical stability. At 170 °C, a pronounced increase in strain was observed, accompanied by a slower and less complete recovery. These trends are consistent with the T-activated nature of the disulfide exchange mechanism and align well with experimental creep behavior.

### Degradability of EPD-2-CF composites

**Scheme 2:**
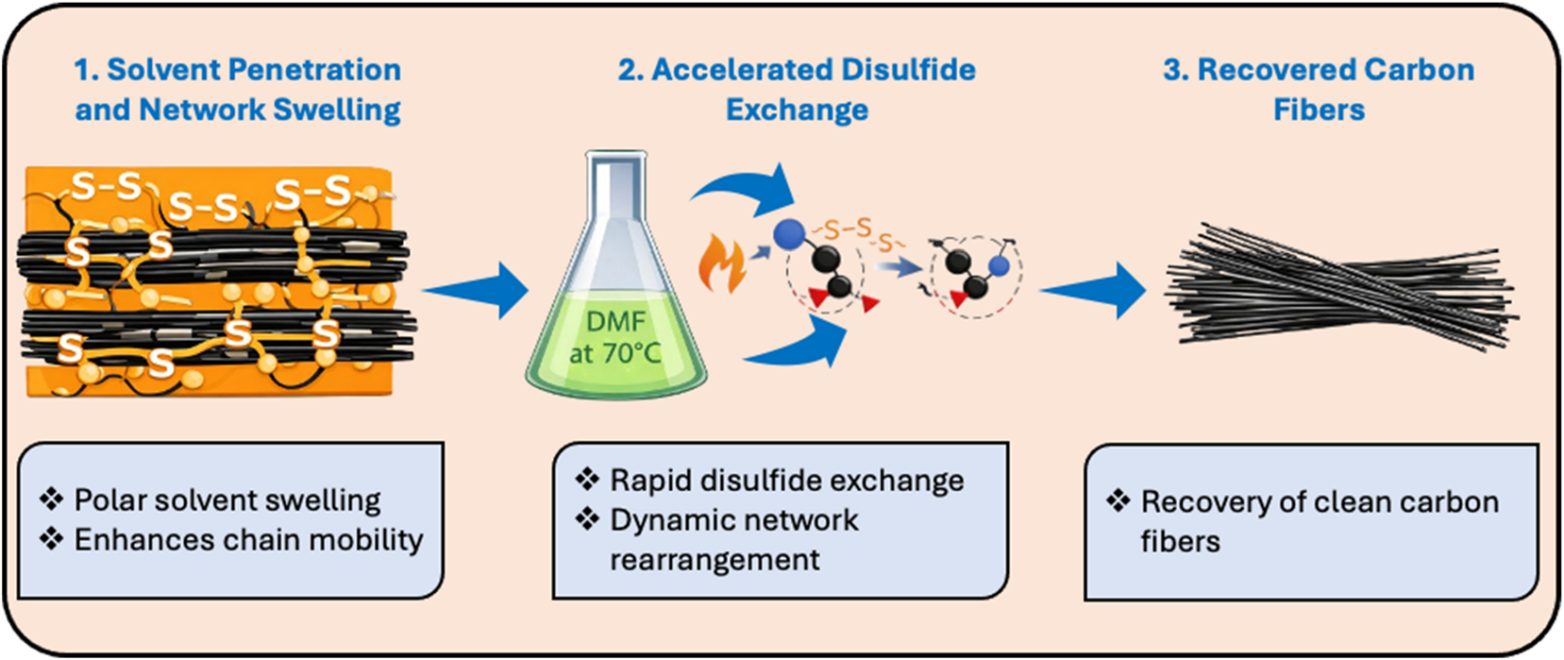
Schematic illustration of DMF-assisted disulfide bond exchange and matrix disintegration.

The vitrimer network contains reversible disulfide crosslinks that enable bond rearrangement through dynamic exchange reactions. Upon exposure to DMF at 70 °C, solvent molecules penetrate and swell the EPD-2 vitrimer matrix, weakening intermolecular interactions and facilitating disulfide bond exchange and network disintegration. As a result, the crosslinked matrix gradually breaks down, allowing the recovery of carbon fibers (CFs) from the EPD-2-CF composites within 4 h. The Scheme 2 illustrates the degradation process. The interfacial interaction between carbon fibers and the epoxy matrix plays a crucial role in determining the mechanical performance of fiber-reinforced composites, as it governs stress transfer between the matrix and the reinforcing fibers. In the present system, the epoxy vitrimer matrix is expected to provide adequate interfacial adhesion with the carbon fiber surface through physical interactions and possible surface functional groups. Although detailed interfacial characterization such as interfacial shear strength measurements was not the primary focus of this study, the recyclability of the vitrimer matrix enables efficient separation and recovery of carbon fibers while preserving their structural integrity, which is important for potential reuse in composite applications.

**Fig. 8:**
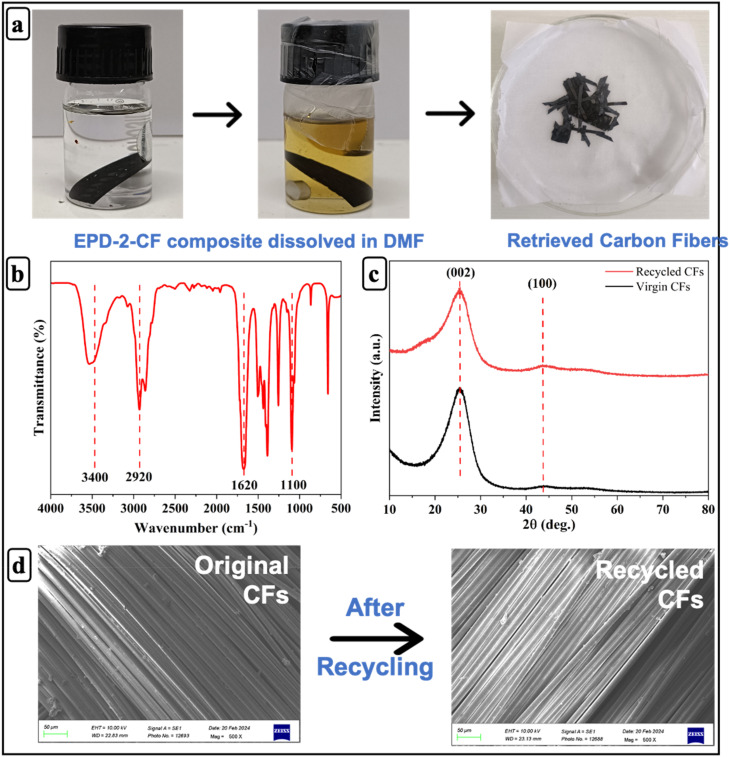
(a) Photographs of the degradability process of EPD-2-CF vitrimer composite; (b) FT-IR spectra of EPD-2-CF degraded product; (c) XRD; (d) SEM of original and recycled carbon fibers.

The photographs in Fig. 8(a) illustrate the degradation process, where the solution progressively darkens due to dissolution of the polymer matrix. The FTIR spectrum of the recovered carbon fibers (Fig. 8(b)) shows a broad absorption band around ∼3400 cm^−1^, corresponding to –OH stretching vibrations associated with surface hydroxyl groups generated during chemical degradation of the matrix. The weak bands near ∼2920 cm^−1^ correspond to C–H stretching vibrations originating from minor residual organic fragments. The peak around ∼1620 cm^−1^ is attributed to C=C stretching of the graphitic carbon structure, while the band near ∼1100 cm^−1^ corresponds to C–O stretching, indicating the presence of oxygen-containing functional groups on the fiber surface.^74^ The XRD patterns of virgin and recycled carbon fibers (Fig. 8(c)) exhibit a prominent diffraction peak at ∼26° corresponding to the (002) plane of graphitic carbon, confirming the characteristic crystalline structure of carbon fibers. A weaker peak around ∼43° corresponding to the (100) plane is also observed. The similarity of diffraction peaks between virgin and recycled fibers indicates that the graphitic crystalline structure remains largely preserved after the recycling process.^75^ Furthermore, SEM images (Fig. 8(d)) reveal that the recycled fibers retain smooth surface morphology comparable to the original CFs, confirming that the recycling process does not adversely affect fiber integrity.

To highlight the performance of the developed vitrimer system, a comparison with previously reported disulfide-based vitrimer networks is presented in Table 3. The obtained thermal stability, activation energy, and recyclability demonstrate that the present system exhibits comparable or improved performance relative to reported vitrimer materials.

**Table 3:** Comparison of the present vitrimer system with reported disulfide-based vitrimer networks

Property	Reported vitrimer system	References	This work
Glass transition temperature (*T*_g_)	147 °C	34	64 °C
Thermal stability (*T*_d5%_)	350 °C	76	396 °C
Activation energy (*E*_a_)	46.8 kJ mol^−1^	66	48 kJ mol^−1^
Storage modulus	33 GPa	42	55 GPa
Recyclability	Reprocessable through dynamic disulfide exchange	22	Matrix degradation and CF recovery using DMF

## Conclusions

In this study, we successfully synthesized and characterized recyclable carbon fiber-reinforced vitrimer composites using DTBA as the dynamic curing agent. The optimized EPD-2 vitrimer matrix exhibited superior thermal stability (*T*_d5%_ of 396 °C), high mechanical strength (290 MPa), and excellent self-healing efficiency (91%) due to the dynamic disulfide bond exchange mechanism. Moreover, the EPD-2-CF composite exhibited exceptional creep recovery and thermoforming capabilities, which can be attributed to the rapid disulfide bond rearrangements that occur at elevated temperatures.

MD simulations aligned well with experimental results, accurately capturing the elastic behavior of vitrimer. The predicted Young's modulus of 2.74 GPa closely matched the experimental value of 2.69 GPa. Creep simulations showed T-dependent strain and partial recovery, supporting the role of dynamic disulfide exchange in stress relaxation and reprocessability.

Furthermore, the developed vitrimer composite exhibited rapid degradation under mild conditions (DMF at 70 °C), enabling the efficient recovery of high-value carbon fibers. The recycled fibers maintained their structural integrity and mechanical performance, as verified by FTIR, XRD, and SEM analyses. Overall, the incorporation of DTBA in epoxy vitrimer matrices presents a promising avenue for developing high-performance, recyclable CFRPs.

Future work will focus on optimizing the recycling process, investigating the long-term stability of the vitrimer network over multiple reprocessing cycles, and evaluating solvent recovery and process scalability for potential industrial applications.

## Author contributions

Harsh Sharma: conceptualization, investigation, data curation, validation, writing – original draft. Nehal Kaushik: conceptualization, formal analysis, validation, writing – review and editing. Songchang Liu: conceptualization, software, formal analysis, visualization, writing – original draft. Anand Rajkamal: conceptualization, formal analysis, software, validation, writing – original draft. Nanda Gopal Sahoo: conceptualization, funding acquisition, project administration, resources, supervision, writing – review and editing. Gun Jin Yun: conceptualization, funding acquisition, project administration, resources, supervision, writing – review and editing. Sravendra Rana: conceptualization, funding acquisition, methodology, project administration, resources, supervision, validation, writing – review and editing.

## Conflicts of interest

There are no conflicts to declare.

## Supplementary Material

RA-016-D6RA00851H-s001

## Data Availability

The data supporting this article have been included as part of the supplementary information (SI), and the file for the same has been uploaded with the submission. Supplementary information is available. See DOI: https://doi.org/10.1039/d6ra00851h.

## References

[cit1] Lefeuvre A., Garnier S., Jacquemin L., Pillain B., Sonnemann G. (2019). Resour., Conserv. Recycl..

[cit2] Dannemand Andersen P., Borup M., Krogh T. (2007). Int. J. Technol. Policy Manag..

[cit3] Sardon H., Dove A. P. (2018). Science.

[cit4] Sharma H., Bender M., Kim G., Lee D., Caglayan C., Schlögl S., Yun G. J., Kumar A., Rana S. (2024). J. Appl. Polym. Sci..

[cit5] Sharma H., Bijalwan V., Mourad A.-H. I., Kumar A., Schlögl S., Rana S. (2024). 0. J. Reinf. Plast. Compos..

[cit6] Wang S., Ma S., Li Q., Xu X., Wang B., Yuan W., Zhou S., You S., Zhu J. (2019). Green Chem..

[cit7] Palmer J., Ghita O. R., Savage L., Evans K. E. (2009). Composites, Part A.

[cit8] Wang Y., Cui X., Ge H., Yang Y., Wang Y., Zhang C., Li J., Deng T., Qin Z., Hou X. (2015). ACS Sustain. Chem. Eng..

[cit9] Cicala G., Pergolizzi E., Piscopo F., Carbone D., Recca G. (2018). Composites, Part B.

[cit10] Iwaya T., Tokuno S., Sasaki M., Goto M., Shibata K. (2008). J. Mater. Sci..

[cit11] Åkesson D., Foltynowicz Z., Christéen J., Skrifvars M. (2012). J. Reinf. Plast. Compos..

[cit12] Oliveux G., Dandy L. O., Leeke G. A. (2015). Prog. Mater. Sci..

[cit13] Zou Z., Zhu C., Li Y., Lei X., Zhang W., Xiao J. (2018). Sci. Adv..

[cit14] Snyder R. L., Fortman D. J., De Hoe G. X., Hillmyer M. A., Dichtel W. R. (2018). Macromolecules.

[cit15] Rowan S. J., Cantrill S. J., Cousins G. R. L., Sanders J. K. M., Stoddart J. F. (2002). Angew. Chem., Int. Ed..

[cit16] Montarnal D., Capelot M., Tournilhac F., Leibler L. (2011). Science.

[cit17] Sharma H., Krishnakumar B., Dickens T. J., Yun G. J., Kumar A., Rana S. (2023). Heliyon.

[cit18] Krishnakumar B., Sanka R. V. S. P., Binder W. H., Parthasarthy V., Rana S., Karak N. (2020). Chem. Eng. J..

[cit19] Krishnakumar B., Pucci A., Wadgaonkar P. P., Kumar I., Binder W. H., Rana S. (2022). Chem. Eng. J..

[cit20] Sharma H., Rana S., Singh P., Hayashi M., Binder W. H., Rossegger E., Kumar A., Schlögl S. (2022). RSC Adv..

[cit21] Shaukat U., Sölle B., Rossegger E., Rana S., Schlögl S. (2022). Polymers.

[cit22] Bohra B. S., Singh P., Rana A., Sharma H., Arya T., Pathak M., Chaurasia A., Rana S., Sahoo N. G. (2023). Compos. Sci. Technol..

[cit23] Bijalwan V., Rana S., Yun G. J., Singh K. P., Jamil M., Schlögl S., Bijalwan V., Rana S., Yun G. J., Singh K. P. (2024). Polym. Rev..

[cit24] Singh P., Binder W. H., Kumar P., Patel R., Yun G. J., Rana S. (2024). ACS Appl. Mater. Interfaces.

[cit25] Krishnakumar B., Prasanna Sanka R. V. S., Binder W. H., Park C., Jung J., Parthasarthy V., Rana S., Yun G. J. (2020). Composites, Part B.

[cit26] Denissen W., De Baere I., Van Paepegem W., Leibler L., Winne J., Du Prez F. E. (2018). Macromolecules.

[cit27] Tian P. X., Li Y. D., Hu Z., Zeng J. B. (2024). Mater. Today Chem..

[cit28] Giebler M., Sperling C., Kaiser S., Duretek I., Schlögl S. (2020). Polymers.

[cit29] Dutta K., Karak N. (2024). Polym. Adv. Technol..

[cit30] Kim G., Caglayan C., Yun G. J. (2022). ACS Omega.

[cit31] Sanka R. V. S. P., Rana S., Singh P., Mishra A. K., Kumar P., Singh M., Sahoo N. G., Binder W. H., Yun G. J., Park C. (2022). Soft Matter.

[cit32] Park C., Kim G., Jung J., Krishnakumar B., Rana S., Yun G. J. (2020). Polymer.

[cit33] Ruiz de Luzuriaga A., Martin R., Markaide N., Rekondo A., Cabañero G., Rodríguez J., Odriozola I. (2016). Mater. Horiz..

[cit34] Si H., Zhou L., Wu Y., Song L., Kang M., Zhao X., Chen M. (2020). Composites, Part B.

[cit35] Aranberri I., Landa M., Elorza E., Salaberria A. M., Rekondo A. (2021). Polym. Test..

[cit36] Barnett P. R., Brackenridge J. A., Advincula A. A., Taussig L. A., Nepal D. (2024). Composites, Part B.

[cit37] Nartam S., Rautela V., Budhe S., Paul J., de Barros S. (2024). Appl. Mech..

[cit38] Hong J., Hong Y., Jeong J., Oh D., Goh M. (2023). ACS Sustain. Chem. Eng..

[cit39] Basu A., Parasuram S., H S., Salvi A. S., Kumar S., Bose S. (2025). ACS Appl. Polym. Mater..

[cit40] Fang M., Liu X., Feng Y., Huang M., Liu C., Shen C. (2024). Compos. Sci. Technol..

[cit41] Luo C., Liu X., Chen Z., Wang Y., He S., Wang J., Li Q., Ruan J., Lin J. (2024). ACS Appl. Polym. Mater..

[cit42] Sharma H., Bijalwan V., Rana S. (2025). Nanoscale Adv..

[cit43] Di Mauro C., Genua A., Mija A. (2021). Polymers.

[cit44] Huang X., Ding C., Wang Y., Zhang S., Duan X., Ji H. (2024). ACS Appl. Mater. Interfaces.

[cit45] Di Mauro C., Genua A., Mija A. (2020). Mater. Adv..

[cit46] Nartam S., Budhe S., Paul J. (2024). Mater. Sci. Forum.

[cit47] Wang Z., Wagner R. J., Chen T., Shah S. P., Maiaru M., Silberstein M. N. (2025). Int. J. Solids Struct..

[cit48] Sharma H., Patel R., Rana S. (2025). ChemistrySelect.

[cit49] Plimpton S. (1995). J. Comput. Phys..

[cit50] Park C., Kim G., Jung J., Krishnakumar B., Rana S., Yun G. J. (2020). Polymer.

[cit51] Rahman R., Haque A. (2013). Composites, Part B.

[cit52] Spreiter Q., Walter M. (1999). J. Comput. Phys..

[cit53] Xiao Y., Xian G. (2018). Polym. Compos..

[cit54] Li X., Zhang X., Chen J., Huang L., Lv Y. (2022). Mater. Today Commun..

[cit55] Park C., Yun G. J. (2018). J. Appl. Mech..

[cit56] Li X., Zhang X., Chen J., Huang L., Lv Y. (2021). Mater. Today Commun..

[cit57] Tsai D. H. (1979). J. Chem. Phys..

[cit58] Martinez-Diaz D., Cortés A., Jiménez-Suárez A., Prolongo S. G. (2022). ACS Appl. Polym. Mater..

[cit59] Denissen W., Winne J. M., Du Prez F. E. (2016). Chem. Sci..

[cit60] Kaiser S., Wurzer S., Pilz G., Kern W., Schlögl S. (2019). Soft Matter.

[cit61] Xu Y., Dai S., Bi L., Jiang J., Zhang H., Chen Y. (2022). Chem. Eng. J..

[cit62] Yang Z., Peng H., Wang W., Liu T. (2010). J. Appl. Polym. Sci..

[cit63] Fernandes S., Correia S., Matos I., Marques M. M., Rana S., Kumar B., Gupta M. K., Singh R. P. (2007). J. Appl. Polym. Sci..

[cit64] Azcune I., Odriozola I. (2016). Eur. Polym. J..

[cit65] Capelot M., Unterlass M. M., Tournilhac F., Leibler L. (2012). ACS Macro Lett..

[cit66] Parameswaran B., Pal T. S., Singha N. K. (2025). RSC Appl. Polym..

[cit67] Verdugo P., Santiago D., De la Flor S., Serra À. (2024). ACS Sustain. Chem. Eng..

[cit68] Gu S., Xu S., Lu J., Pu X., Ren Q., Xiao Y., Wang Y., Chen L. (2023). EcoMat.

[cit69] Liu H., Liu C., Liu Y., Jiang Y., Li X., Bai Y. (2024). Polym. Test..

[cit70] Pepels M., Filot I., Klumperman B., Goossens H. (2013). Polym. Chem..

[cit71] Pimenta S., Pinho S. T. (2011). Waste Manag..

[cit72] Guo X., Liu F., Lv M., Chen F., Gao F., Xiong Z., Chen X., Shen L., Lin F., Gao X. (2022). Polymers.

[cit73] Yao H., Xu X., Chen Z., Li K., Wang T., Pei S., Fu J., Wang J. (2025). RSC Adv..

[cit74] Tian Z., Wang Y., Hou X. (2022). New Carbon Mater..

[cit75] Wu J., Pan Y., Ruan Z., Zhao Z., Ai J., Ban J., Jing X. (2022). Front. Mater..

[cit76] Liu H., Sun Z., Wei L., Liu Y., Zhou S., Ge Q., Liu C., Li X. (2023). Polym. Test..

