# Supplementary material for: Reprocessable carbon fiber composites via disulfide-exchange epoxy vitrimers: experimental and molecular simulation insights
Source: RSC Adv. 2026 Mar 31;16(19):17480–96. doi: 10.1039/d6ra00851h (PMC13037380; doi:10.1039/d6ra00851h)
Supplement: RA-016-D6RA00851H-s001 [file RA-016-D6RA00851H-s001.pdf]

Supplementary Information (SI) for RSC Advances.
This journal is © The Royal Society of Chemistry 2026

Supplementary Information

Reprocessable Carbon Fiber Composites via Disulfide-Exchange Epoxy Vitrimers: Experimental and Molecular Simulation Insights

Harsh Sharma[a,b] *, Nehal Kaushik*[a]*, Songchang Liu*[c]*, Rajkamal Anand*[d]*, NandGopal Sahoo*[e]*,*
Gun Jin Yun[c,d,]***, *Sravendra Rana*[a,]***

[a]*UPES, School of Engineering, Energy Acres, Bidholi, Dehradun, Uttarakhand 248007, India*

[b]*TIET-VT, Centre of Excellence in Emerging Materials (CEEMS), Thapar Institute of Engineering and Technology, Patiala, Punjab, 147004, India*

[c]*Department of Aerospace Engineering, Seoul National University, Seoul, 08846, South Korea*

[d]*Institute of Advanced Aerospace Technology, Seoul National University, Gwanak-gu Gwanak-ro 1, Seoul 08826, Republic of Korea*

[e]*Prof. Rajendra Singh Nanoscience and Nanotechnology Centre, Department of Chemistry, D.S.B. Campus, Kumaun University, Nainital 263001, Uttarakhand, India*

Email: srana@ddn.upes.ac.in (SR); gunjin.yun@snu.ac.kr (GJY)

Rationalizing DTBA Concentration in Aromatic and Bio-Based Epoxy Systems:

It is worth mentioning that conventional epoxy resins such as diglycidyl ether of bisphenol-A (DGEBA) possess a rigid aromatic backbone and a high concentration of uniformly distributed epoxy groups, which contribute to a dense and homogeneous crosslinked network. This structural regularity enhances the reactivity between epoxy and carboxylic acid functionalities, allowing efficient dynamic bond formation even at low concentrations of crosslinkers such as 2,2'-dithiodibenzoic acid (DTBA). The well-defined molecular architecture of DGEBA facilitates efficient curing and vitrimer-like behavior without requiring excess DTBA.

In contrast, acrylated epoxidized soybean oil (AESO), a bio-based epoxy precursor, features an aliphatic and partially branched structure with inherent flexibility. Additionally, the acrylation of epoxidized soybean oil leads to a partial reduction in epoxy group density, as some oxirane functionalities are consumed or rendered sterically less accessible during the functionalization process. This results in a comparatively lower concentration of effective reactive sites for network formation. Therefore, in our work, a consistent 2 wt% DTBA concentration combined with 10 wt% tin(II) 2-ethylhexanoate catalyst has been employed across varying DGEBA/AESO ratios. This formulation is designed to accommodate the structural characteristics of both aromatic and bio-based epoxy-acrylate matrices while preserving the dynamic covalent behavior essential for vitrimer performance.

Preparation and Solubility Analysis of Epoxy Vitrimers with Higher DTBA Concentration:

To address concerns regarding vitrimer formation at low 2,2'-dithiodibenzoic acid (DTBA) concentrations, additional epoxy thermosets were prepared using higher DTBA loadings of 5, 10, and 15 wt%. These formulations were synthesized following the same procedure as the primary system. However, the curing behavior of high-DTBA samples deviated significantly, suggesting that excessive disulfide content may interfere with network formation or result in inhomogeneities.

Curing Behavior of High-DTBA Formulations:

Unlike the successfully cured vitrimer networks at 2 wt% DTBA, the samples with 5, 10, and 15 wt% DTBA exhibited incomplete or failed curing. The excess DTBA disrupted the crosslinking process due to an excessive number of thiol-disulfide interactions, which likely interfered with the network formation of diglycidyl ether of bisphenol A (DGEBA). This resulted in either partial gelation or the complete inhibition of the curing process, leading to mechanically weak or uncured materials. Figure S1 shows the molded samples before solubility test.

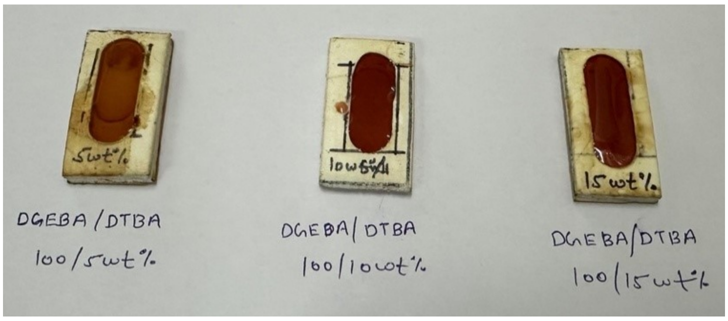

Figure S1. *Epoxy samples prepared with 5, 10 and 15 wt% DTBA concentration.*

Solubility Test of High-DTBA Formulations:

To confirm the absence of a stable crosslinked network in these samples, a solubility test was conducted. Each sample was immersed in acetone at room temperature, and all three compositions (5, 10, and 15 wt% DTBA) completely dissolved within two minutes. This rapid dissolution indicates the lack of a sufficiently crosslinked network, confirming that excessive DTBA hinders proper vitrimer formation. Figure S2 demonstrates the dissolution behavior of high-DTBA samples in acetone.

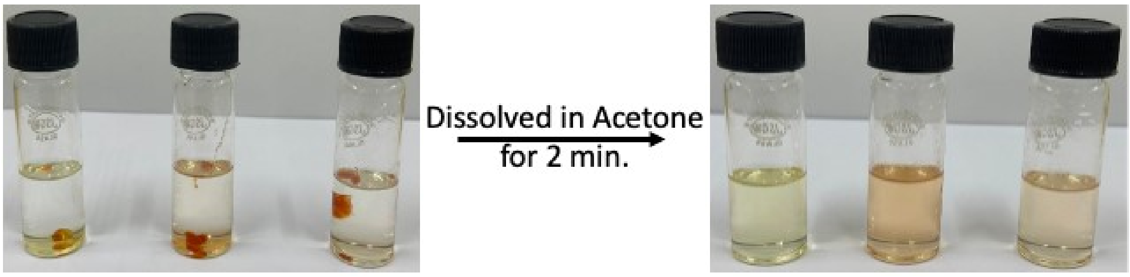

Figure S2. *Dissolved epoxy samples of 5, 10, and 15 wt% DTBA concentration.*
